# Osmotic demyelination without hyponatremia: the role of glycemic variability in a complex patient with diabetes

**DOI:** 10.1093/omcr/omag138

**Published:** 2026-07-27

**Authors:** Juliana Pineda-Ortega, María Juliana Soto-Chavez, Ana María Gómez

**Affiliations:** Departamento de Medicina Interna, Hospital Universitario San Ignacio, Carrera 7 No. 40-62, Bogotá, Colombia; Facultad de Medicina, Departamento de Medicina Interna, Pontificia Universidad Javeriana, Carrera 7 No. 40-62, Bogotá Colombia; Departamento de Medicina Interna, Hospital Universitario San Ignacio, Carrera 7 No. 40-62, Bogotá, Colombia; Facultad de Medicina, Departamento de Medicina Interna, Pontificia Universidad Javeriana, Carrera 7 No. 40-62, Bogotá Colombia; Hospital Universitario San Ignacio, Unidad de Endocrinología, Carrera 7 No. 40-62, Bogotá, Colombia; Departamento de Medicina Interna, Hospital Universitario San Ignacio, Carrera 7 No. 40-62, Bogotá, Colombia; Facultad de Medicina, Departamento de Medicina Interna, Pontificia Universidad Javeriana, Carrera 7 No. 40-62, Bogotá Colombia; Hospital Universitario San Ignacio, Unidad de Endocrinología, Carrera 7 No. 40-62, Bogotá, Colombia

**Keywords:** central pontine myelinolysis, glycemic variability, osmotic demyelination, type 1 diabetes mellitus

## Abstract

Osmotic demyelination syndrome (ODS) is classically associated with rapid correction of hyponatremia; however, non-sodium osmotic shifts remain underrecognized. We report a young man with poorly controlled type 1 diabetes and extreme glycemic variability (13–486 mg/dL), admitted with cardiorenal syndrome requiring dialysis, who developed progressive neurological deficits. Brain MRI demonstrated findings consistent with extrapontine ODS despite stable sodium levels. Alternative etiologies were excluded, supporting glycemic variability and osmotic instability as key contributors, potentially exacerbated by dialysis disequilibrium. This case expands the pathophysiological spectrum of ODS and highlights glycemic variability as a clinically relevant, yet overlooked, risk factor.

## Introduction

According to the International Diabetes Federation (IDF), 10.5% of people aged 20–79 years worldwide have diabetes, with approximately 10% of these cases being type 1 diabetes mellitus (DMT1) [1]. Proper metabolic control has been shown to reduce microvascular and macrovascular complications associated with both type 1 and type 2 diabetes [2]. Glycemic variability is defined as acute glycemic fluctuations, both upward (postprandial hyperglycemia or hypoglycemia correction) and downward (from excessive hypoglycemic treatment), which lead to increased oxidative stress, endothelial dysfunction, and vascular damage [2]. Clinically, this translates to an increase in microvascular and macrovascular complications, a higher risk of hypoglycemia, and even mortality [3].

Osmotic demyelination syndrome (ODS), also known as central pontine myelinolysis (CPM), is syndrome mostly associated with the rapid correction of hyponatremia. However, other triggering factors such as hypernatremia, hypokalemia, magnesium level alterations, lithium treatment, and, less commonly, glycemic variability have been proposed [4]. Although evidence remains limited, isolated case reports have documented ODS in patients with DMT1 in the absence of dyselectrolytemia, suggesting glucose driven osmotic shifts may be sufficient to trigger demyelination [5]. Given the high prevalence of diabetes, it is important to consider other potential complications resulting from poor disease control, such as glycemic variability, and its potential association with neurological alterations such as ODS. We present the case of a young man with poorly controlled diabetes, high glycemic variability, and multiple hypoglycemic episodes who developed neurological deterioration with imaging findings suggestive of CPM.

## Case report

We report the case of a 24-year-old man with a history of type 1 diabetes mellitus diagnosed at the age of seven, with no proper medical follow-up and poor outpatient metabolic control (glycated hemoglobin, 10.2%), who denied any history of alcohol abuse, illicit drug use or smoking. He was admitted because of acute heart failure syndrome (NT-proBNP 21 892 pg/mL) associated with cardiorenal syndrome, requiring emergent renal replacement therapy (creatinine 16 mg/dL, BUN 136 mg/dL, metabolic acidosis, signs of central and peripheral volume overload, mild hyperkalemia), and uncontrolled hypertension. He was transferred to the intensive care unit, where hemodialysis and vasodilators were initiated. Echocardiography showed a left ventricular ejection fraction of 65% with minimal pericardial effusion.

During his ICU stay, he exhibited extreme glycemic variability from 13 to 486 mg/dL (Fig. 1) with episodes of severe hypoglycemia and postprandial hyperglycemia, despite transitioning to a second-generation insulin analog (degludec) and daily insulin titration with endocrinology support.

Further studies documented severe bilateral proliferative retinopathy, indicating poor visual prognosis. He subsequently developed left peripheral facial paralysis, attributed to diabetic cranial mononeuropathy, a recognized complication in poorly controlled diabetes with established microangiopathy [6]. It was managed with protective measures and physiotherapy, and corticosteroids were withheld to avoid glycemic destabilization. Additionally, he experienced somnolence, postural instability, and horizontal diplopia due to limited abduction of the left eye, scanning dysarthria, and lower limb paraparesis (muscle strength 4/5), predominantly on the left side. Cranial CT revealed reduced density in the corpus callosum and poorly defined hypodensity in the right internal capsule. Brain MRI revealed increased signal intensity in the corpus callosum and deep posterior frontal white matter lesions with marked diffusion restriction without gadolinium enhancement (Fig. 2). The imaging findings suggested atypical posterior reversible encephalopathy syndrome (PRES) versus extrapontine ODS, excluding abrupt hyponatremia correction (sodium levels between 132 and 137 mmol/L during hospitalization). The lesions were considered multifactorial, particularly associated with long-standing glycemic variability, along with a potential post-dialysis disequilibrium syndrome (initial BUN, 132 mg/dL, post-dialysis 76.7 mg/dL) and poorly controlled hypertension upon admission.

**Figure 1:**
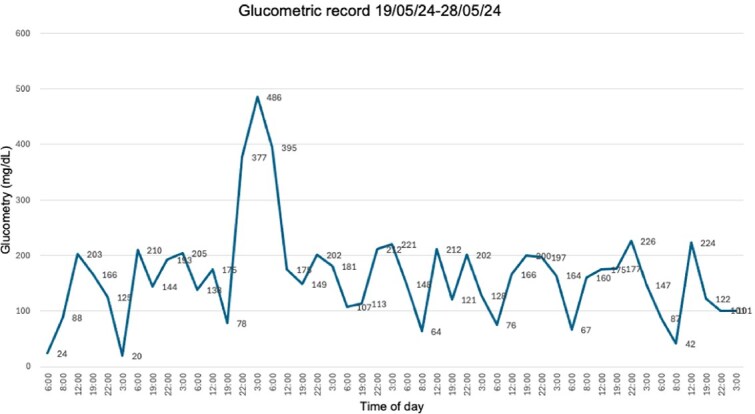
Glucose readings recorded from may 19 to may 28, 2024, demonstrating the patient's high glycemic variability.

**Figure 2:**
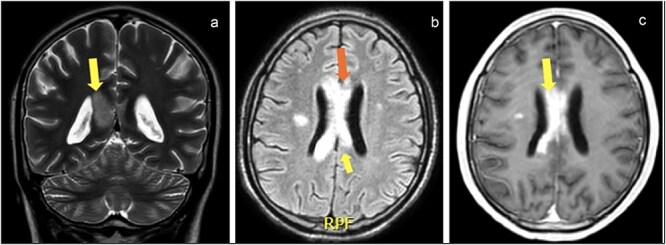
Brain MRI with gadolinium. Hyperintense lesions on T2 (a) and FLAIR (b) sequences, extensively involving the corpus callosum, predominantly in its right parasagittal fibers (yellow arrows), and to a lesser extent in the deep white matter of the posterior aspect of both frontal lobes (orange arrow), now showing enhancement after intravenous gadolinium injection (c). These findings may correspond to subacute ischemia in the context of atypical PRES versus predominantly extrapontine osmotic demyelination.

**Figure 3:**
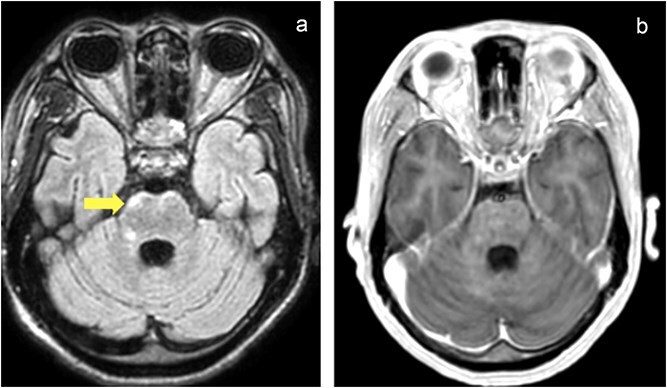
Brain MRI with gadolinium. T2 and FLAIR sequences (a) also show small nonspecific hyperintensities in the periphery of the pons (predominantly on the right side), as indicated by the arrow, and in the anterior left aspect of the pontobulbar junction, without significant diffusion signal restriction and (b) without apparent enhancement after intravenous gadolinium injection. These findings are possibly of the same etiology as those documented in the corpus callosum and posterior frontal lobe, but with different timing.

The patient also experienced recurrent vomiting episodes, increasing somnolence, and orbitofrontal headache; lumbar puncture excluded intracranial hypertension, but showed hyperproteinorrachia and a positive VDRL serology, establishing a diagnosis of neurosyphilis, treated with penicillin, after which the patient was more alert, but the rest of neurological clinical findings remained unchaged. Glycemic variability persisted throughout hospitalization despite recalculated insulin doses and corrections based on carbohydrate counting, correction factors, and carbohydrate-to-insulin ratios. Due to the deterioration of mental status and suspected swallowing dysfunction, enteral nutrition was initiated. Follow-up brain MRI revealed persistent hyperintense lesions on T2 and FLAIR sequences, with gadolinium enhancement and new hyperintense lesions in the brainstem (Fig. 3).

After multidisciplinary assessment (internal medicine, neurology, endocrinology, nephrology, and clinical ethics) the demyelinating lesions were attributed to the combined effect of glycemic variability, severe hypertension, and post-dialysis disequilibrium syndrome. The patient’s long-standing uncontrolled diabetes, end-stage renal disease on dialysis, and establish microvascular complications constituted the full risk factor profile for high glycemic variability. Other causes of hypoglycemia, such as adrenal insufficiency (morning cortisol 5.86 μg/dL), were ruled out. The prognosis was considered poor, with a high likelihood of permanent neurological sequelae.

The patient was discharged for comprehensive rehabilitation, psychiatric evaluation, speech therapy to reassess enteral nutrition, and outpatient diabetes technology follow-up.

## Discussion

This is an unusual case of imaging findings suggestive of central pontine myelinolysis not associated with abrupt correction of hyponatremia in a patient with poorly controlled diabetes, high glycemic variability, and multiple hypoglycemic episodes. ODS has been described in relation to the rapid correction of chronic and severe hyponatremia, often associated with other risk factors such as prolonged fasting and alcoholism [3]. However, ODS has also been recognized in various conditions causing rapid and significant changes in plasma osmolality, even without alterations in sodium homeostasis, such as hemorrhagic or hypoxemic conditions, hypokalemia, and glycemic variability, especially severe hypoglycemia [7]. Demyelination or damage to the myelin sheath is rare, reported in 0.06% of hospitalized patients and in up to 0.4% of admissions with neurological symptoms [3]. This condition arises from rapid osmolar shifts, releasing inorganic and organic osmolytes (such as chloride and myoinositol, respectively), producing cellular contraction and apoptosis, predominantly affecting the extrapontine regions [3, 8].

In this patient, sustained hyperglycemia with abrupt fluctuations produced rapid shifts away from a hyperosmolar state. Other etiologies of the neuroimaging and clinical findings including chronic alcoholism, lithium toxicity, refeeding syndrome, severe malnutrition and primary demyelinating disease were excluded, while dialysis disequilibrium syndrome was identified as a probable exacerbating factor.

## Conclusion

This case supports the emerging concept that ODS can occur in the absence of dysnatremia when rapid plasma osmolarity shifts arise from other solutes, particularly glucose. In patients with diabetes with high glycemic variability, microvascular complications and concurrent uremic or dialysis-related osmotic stress, clinicians should maintain a low threshold for neuroimaging when unexplained neurological deficits arise. Thus, it is essential to avoid abrupt corrections of any osmotically active disturbances to prevent neurological changes that can severely impact patients' quality of life and prognosis.
